# Magnetic resonance imaging-radioguided occult lesion localization (ROLL) in breast cancer using Tc-99m macro-aggregated albumin and distilled water control

**DOI:** 10.1186/1471-2342-13-33

**Published:** 2013-09-18

**Authors:** Fernanda Philadelpho Arantes Pereira, Gabriela Martins, Maria Julia Gregorio Calas, Maria Veronica Fonseca Torres de Oliveira, Emerson Leandro Gasparetto, Lea Mirian Barbosa da Fonseca

**Affiliations:** 1 Department of Radiology, Federal University of Rio de Janeiro, Rua Prof. Rodolpho Paulo Rocco 255, Cidade Universitária, Rio de Janeiro, RJ 21941-617, Brazil; 2 Department of Breast Imaging, Clínica de Diagnóstico por Imagem (CDPI), Av. Ataulfo de Paiva 669, 2nd floor, Leblon, Rio de Janeiro, RJ 22440-032, Brazil; 3 Department of Magnetic Resonance Imaging, Clínica de Diagnóstico por Imagem (CDPI), Av. Ataulfo de Paiva 669, 2nd floor, Leblon, Rio de Janeiro, RJ 22440-032, Brazil; 4 Department of Nuclear Medicine, Federal University of Rio de Janeiro, Rua Prof. Rodolpho Paulo Rocco 255, Cidade Universitária, Rio de Janeiro, RJ 21941-617, Brazil; 5 Department of Nuclear Medicine, Clínica de Diagnóstico por Imagem (CDPI), Av. Ataulfo de Paiva 669, 2nd floor, Leblon, Rio de Janeiro, RJ 22440-032, Brazil

**Keywords:** Magnetic resonance imaging (MRI), MR-guided interventional procedures, Nuclear medicine, Radioisotopes, Breast cancer

## Abstract

**Background:**

Magnetic resonance imaging (MRI) guided wire localization presents several challenges apart from the technical difficulties. An alternative to this conventional localization method using a wire is the radio-guided occult lesion localization (ROLL), more related to safe surgical margins and reductions in excision volume. The purpose of this study was to establish a safe and reliable magnetic resonance imaging-radioguided occult lesion localization (MRI-ROLL) technique and to report our initial experience with the localization of nonpalpable breast lesions only observed on MRI.

**Methods:**

Sixteen women (mean age 53.2 years) with 17 occult breast lesions underwent radio-guided localization in a 1.5-T MR system using a grid-localizing system. All patients had a diagnostic MRI performed prior to the procedure. An intralesional injection of Technetium-99m macro-aggregated albumin followed by distilled water was performed. After the procedure, scintigraphy was obtained. Surgical resection was performed with the help of a gamma detector probe. The lesion histopathology and imaging concordance; the procedure’s positive predictive value (PPV), duration time, complications, and accuracy; and the rate of exactly excised lesions evaluated with MRI six months after the surgery were assessed.

**Results:**

One lesion in one patient had to be excluded because the radioactive substance came back after the injection, requiring a wire placement. Of the remaining cases, there were four malignant lesions, nine benign lesions, and three high-risk lesions. Surgical histopathology and imaging findings were considered concordant in all benign and high-risk cases. The PPV of MRI-ROLL was greater if the indication for the initial MR examination was active breast cancer. The median procedure duration time was 26 minutes, and all included procedures were defined as accurate. The exact and complete lesion removal was confirmed in all (100%) patients who underwent six-month postoperative MRI (50%).

**Conclusions:**

MRI-ROLL offers a precise, technically feasible, safe, and rapid means for performing preoperative MRI localizations in the breast.

## Background

Magnetic resonance imaging (MRI) of the breast has proven to be a valuable complement to the conventional techniques, including mammography, ultrasonography, and physical examination, for breast cancer detection, diagnosis, staging, and treatment follow-up [1]. In addition, MRI is able to detect lesions that are not visible on these conventional techniques in 10-39% of cases [2]. In patients with proven breast cancer, MRI can detect additional ipsilateral cancer sites in 6-34% of cases and unsuspected contralateral cancer in 4-24% of cases [3-5]. However, as the reported specificity of breast MRI (37–97%) is lower than its high sensitivity (94-100%), suspicious lesions detected by MRI must be confirmed histologically [3].

When suspicious, enhancing breast lesions are detected solely with MRI, MRI-guided biopsy techniques are used for accurate sampling of the lesions and for histopathological analysis. MRI-guided tissue sampling of these “MRI-only observed lesions” can be accomplished by needle localization followed by surgical excision, by MRI-guided large-core needle biopsy, or through vacuum biopsy [1,6]. For an interventional procedure to be clinically useful, factors such as safety, accuracy, availability, cost, patient preference, and surgeon’s request should be considered [7]. MRI-guided needle localization is a well-known and widely utilized technique for tissue sampling, especially for breast lesions that are difficult to access [1,6-8].

Until now, MRI-guided needle localization has been performed through the deployment of a wire. While the accuracy of needle and wire placement is important with any means of guidance, it is particularly important for MRI-guided procedures because lesion retrieval cannot be verified with radiography of the lumpectomy specimen, as the lesion is commonly only visible *in vivo* after the intravenous administration of a gadolinium-based contrast material [9]. Although excisional biopsy after MRI-guided wire localization has proven to be a successful method for obtaining adequate material for pathological evaluation, this technique is associated with several challenges apart from the technical difficulties [10-12], including the accordion effect, which leads to a final wire position that is not the ideal after breast decompression; wire displacement and migration; wire breakage; difficulties related to establishing surgical access to the lesion; infection; and bleeding. Nevertheless, its main disadvantage is the high incidence of residual disease (up to 51% at the biopsy site) [12-15].

An alternative to this conventional localization method using a wire is the radio-guided occult lesion localization (ROLL), which consists of an intratumoral injection of Technetium-99m (Tc-99m) macro-aggregated albumin (MAA). On the day of surgery, a portable gamma probe guides the biopsy, providing a practical and precise method for locating the intratumor injection site. In the last years, this technique has been proposed by many different studies as being the best option for the localization of non-palpable breast lesions guided by mammography or ultrasonography [12,16-19], resulting in correct localization in more than 90% of cases. It has also been associated with a higher prevalence of safe surgical margins, improved cosmetic outcome, and less postoperative pain, in addition to reductions in excision volume and more accurate lesion centricity within the surgical specimen [7,12-20].

Although ROLL has been used for more than 10 years for mammography- and ultrasonography-guided localizations, to the best of our knowledge, there is only one study in the literature describing the use of radioactive substances in MRI-guided localizations using a different technique than the one described here [7]. Hence, this study was designed to establish a safe and reliable technique and to report our initial experience with MRI-ROLL of nonpalpable breast lesions only observed on MRI.

### Methods

#### Study population and lesion characteristics

From May 2011 to July 2012, this study prospectively enrolled 16 women with 17 breast lesions. One lesion in one patient was excluded from the study because the procedure was unsuccessful, and a wire was required. As a result, the study included 15 patients (age range, 38–78 years; mean age, 53.3 years) with 16 breast lesions.

All patients had a diagnostic MRI performed prior to the procedure, showing lesions not identified by mammography or ultrasonography, even by “second look” ultrasonography. The size range of these 16 lesions was 0.6-4.0 cm (median, 1 cm). Eleven lesions (68.75%) were located in the left breast, and five lesions (31.25%) were located in the right breast.

The ACR BI-RADS-MRI Lexicon was used to classify the morphological and dynamic characteristics of the lesions [21]. Eight of the 16 lesions (50%) were non-mass enhancement-type lesions, and the remaining eight (50%) were mass-type lesions. From the eight non-mass enhancement lesions, three showed focal distribution, three ductal distribution, one segmental distribution, and one linear distribution. From the eight mass lesions, six had oval shape and smooth margins, one had a lobulated shape and smooth margins, and one had irregular shape and margins. Most lesions had either moderate or early marked enhancement. The delayed enhancement pattern analyzed in the mass lesions showed plateau curves in three out of the eight lesions and washout curves in the remaining five lesions. The BI-RADS classifications were BI-RADS 4 for 14 of the 16 lesions and BI-RADS 3, which signifies a likely benign lesion, for 2 of the 16 lesions.

Indications for the previous MRI examinations were problem-solving in nine cases, a present history of ipsilateral or contralateral breast cancer in three cases, a previous personal history of breast cancer in one case, search for an occult primary tumor in one patient with an altered axillary lymph node, and integrity of breast implants in two cases.

Our institutional review (CEP HUCFF/FM) board approved the study, and all patients gave their informed consent.

#### MRI-ROLL technique

All MRI-ROLL were performed by one of three radiologists (FPAP, GM, MJGC) who were experts in breast imaging, including MRI-guided breast procedures, using a 1.5-T MR System (Signa Excite HD, GE Healthcare, Milwaukee, WI) with the patient positioned prone in a dedicated 8-channel breast coil. The breast undergoing localization was placed in the coil using a grid-localizing system. First, the medial aspect of the breast was positioned flush against a compression plate or a grid, depending on whether the access to the lesion was lateral or medial, respectively. A lateral grid or compression plate, also depending on whether the access was lateral or medial, respectively, was then firmly adjusted to fully compress and immobilize the breast. A vitamin E capsule was used as a fiducial marker and was taped to the grid over the expected lesion site, which was determined based on review of the diagnostic MR images.

First, a localizing sequence was acquired, and the volume of interest was selected to include the compression device and the vitamin E marker. Then, a sagittal T1-weighted 3D fat-suppressed gradient-echo sequence (flip angle, 15°; bandwidth, 41.67 MHz; matrix size, 220 × 220; field of view, 220 mm; number of excitations, 1; slice thickness, 2 mm; intersection gap, 0 mm) was repeated before and after the rapid bolus injection of 0.1 mmol/L of gadoterate meglumine (Dotarem, Guerbet, Roissy, France) per kilogram of body weight, followed by 20 mL of saline, until the enhancing lesion was visualized. The acquisition time, which was approximately 1 min per sequence, varied depending on the size of the breast and the area covered.

The images were reviewed. A cursor was placed over the lesion on the monitor, and its relationship to the skin surface and the vitamin E marker was determined by manually scrolling through sequential sagittal slices. The grid of the compression device was evident as low-signal-intensity lines on the skin surface due to pressure indentation. The plastic of the compression device was not visible on MRI. The vitamin E capsule was identified as an area of high signal intensity on the skin surface. The skin entry site was determined based on visual assessment of the location of the lesion with respect to the grid lines using the vitamin E capsule as a guide. The depth of the lesion from the skin surface was calculated as the difference between the depth of the skin surface and the depth of the sagittal slice containing the lesion.

After calculating the entrance site and lesion depth, the patient was withdrawn from the magnet. The skin overlying the lesion was marked, and the skin was cleansed with alcohol and anesthetized with 1–2 mL of 1% lidocaine hydrochloride (Xylocaine, Astra USA, Westborough, MA). A needle guide block (Needle block, In Vivo, Gainesville, FL) was inserted into the grid hole overlying the anesthetized area. A needle guide with 20-gauge holes was used to anchor and stabilize the needle and to allow insertion of the needle in a straight perpendicular fashion, thereby reducing needle angulation during insertion. The MR-compatible needle (20 ga, MRI-compatible needle, EZEM, Westbury, NY) was then placed in the hole of the needle guide closest to the skin marking. We inserted the needle to the desired depth, taking into account the 1.5-cm thickness of the needle guide.

Sagittal and axial T1-weighted 3D fat-suppressed gradient-echo sequences were then obtained to document the location of the needle, with the desired depth of the tip optimally positioned within the lesion. The needle was evident as a low-signal-intensity structure with an adjacent susceptibility artifact. If the needle was too deep or too superficial, adjustments were made.

When the needle tip was within the lesion or at least within 5 mm of the lesion, a sagittal fat-suppressed T2-weighted fast spin-echo sequence (repetition time/echo time, 2300/102 ms; bandwidth, 35.7 MHz; matrix size, 256 × 224; field of view, 230 mm; number of excitations, 4; slice thickness, 2 mm; intersection gap, 0 mm; and acquisition time, 1.46 minutes) was repeated before and after the injection of the radioactive substance (Tc-99m MAA). The injected dose was 1 mCi or 37 MBq if the surgery had been performed the same day as the localization or 4 mCi or 148 MBq if the surgery had been performed the day after, followed by the administration of 1 mL of distilled water. In this sequence, the needle is also evident as a low-signal-intensity structure with an adjacent susceptibility artifact. In contrast, the water injected after the radioactive substance is evident as a high-signal-intensity area and confirms the injection of the radioactive material in the proper location within the lesion. The subtraction technique was also used as a control for the radioactive substance injection. The T2-weighted sequence obtained before the radioactive material plus water administration was subtracted from the T2-weighted sequence obtained after the radioactive substance plus water administration, and the high-signal-intensity area from the injected water was better visualized. The needle was then removed.

Immediately after localization, scintigraphy (Ventri, GE Healthcare, Milwaukee, WI) was performed to serve as a control for the presence of the radioactive substance; a two-view mammography (Lorad M-IV, Hologic, Bedford, MA) was also performed to serve as a road map for the surgeon. We were aware that the radioactive substance would not appear on the mammographic images. The report and the images obtained from the MRI localization and from the scintigraphic and mammographic control were sent to the surgeon along with the patient. A six-month follow-up MRI was suggested in the report to confirm lesion removal, mainly for benign cases.

During the surgery, a gamma probe (Johnson & Johnson, New Jersey, NY) was used to locate the maximum radioactive focus and, thus, the lesion. The complete removal of the lesion was confirmed by the absence of radioactivity in the breast tissue and by the presence of safe surgical margins. The excised tissue was sent for histopathological examination.

#### MRI analysis and data collection

Histopathological characteristics of the lesion were determined from the surgical pathology reports and were categorized as benign, malignant, or high-risk. MR imaging and histopathological findings were reviewed and considered concordant if the histopathological findings provided an explanation for the imaging features, particularly in the benign and high-risk lesions. The positive predictive value (PPV) of MRI-ROLL was defined as the number of cancers identified during MRI localization divided by the total number of lesions that had undergone MRI localization. The PPV was also analyzed according to the indication of the previous MRI examination, i.e., the MRI that first showed the lesion.

Breast MRI-ROLL cases were reviewed to assess the procedure time (total magnet time), complication rate, and accuracy. The procedure accuracy was measured by the distance between the needle tip and the target lesion prior to the injection of the radioactive material, by the presence of high-signal-intensity area on T2-weighted sequence and on the subtracted images in the exact location of the lesion after the water administration, and by the presence of the radioactive material in the scintigraphic control. The rate of exactly excised lesions was evaluated on the MRI obtained six months postoperatively.

Data were collected and analyzed on a computerized spreadsheet (Excel, Microsoft, Redmond, WA).

## Results

### Histopathological findings

**Figure 1:**
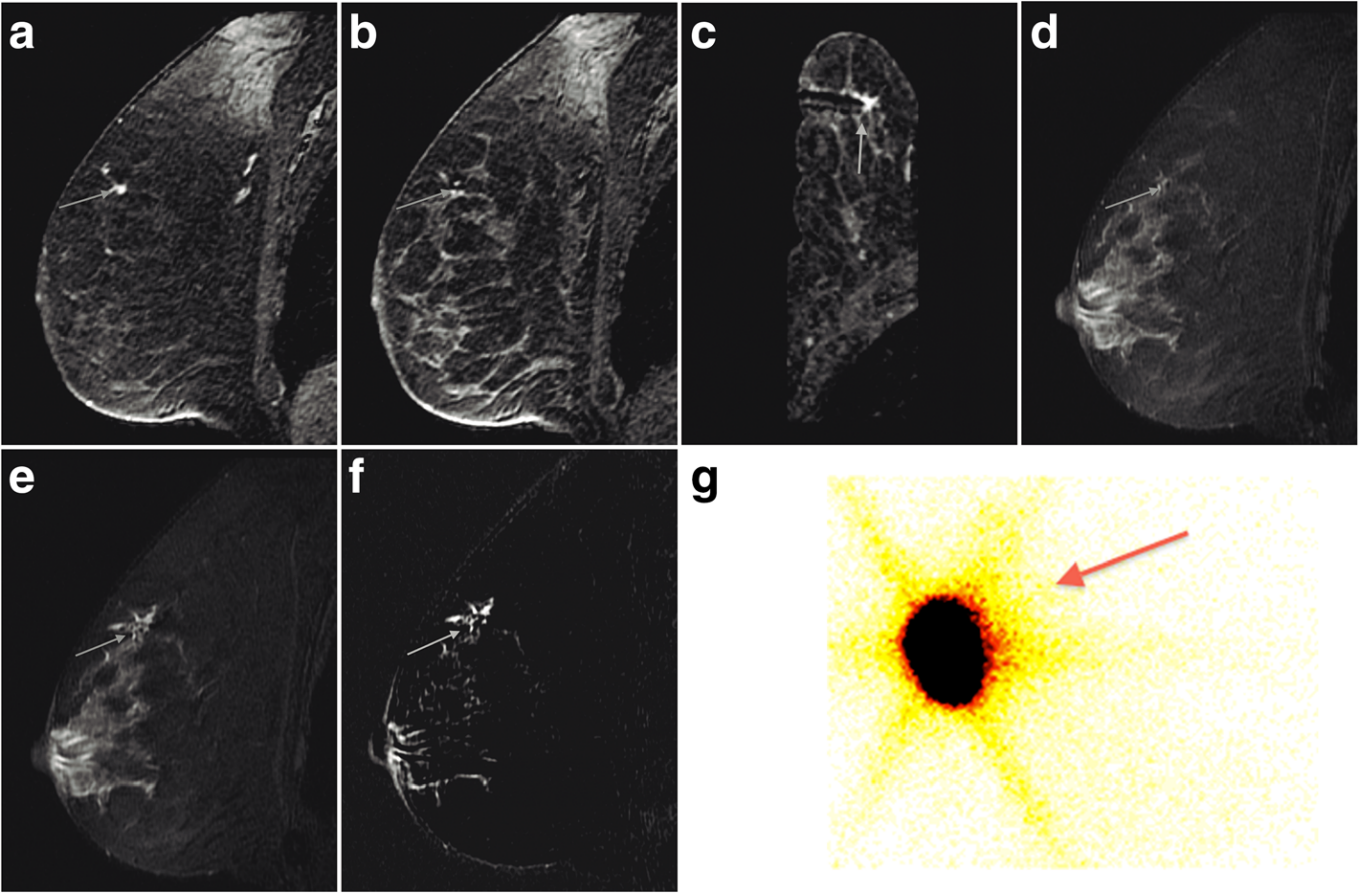
**A 78-year-old woman with invasive ductal carcinoma (IDC) of the right breast. (a)** Sagittal T1-weighted contrast-enhanced sequence shows a regular mass in the upper inner quadrant of the right breast (arrow). **(b)** Sagittal T1-weighted contrast-enhanced sequence reveals the lesion with the needle, low-signal-intensity dot, inside (arrow). **(c)** Axial T1-weighted contrast-enhanced sequence shows the needle tip close to the lesion (arrow) **(d)** Sagittal fat-suppressed T2-weighted sequence shows the exact location of the lesion after correlation with the contrast-enhanced sequences (arrow). **(e)** Sagittal T2-weighted sequence after injection of Tc-99m MAA followed by injection of 1 mL of distilled water reveals a high-signal-intensity area and confirms that the radioactive material is in the exact location of the lesion. **(f)** Sagittal T2-weighted sequence subjected to subtraction technique: the high-signal-intensity area from the water injected is visualized better (arrow). **(g)** Scintigraphic control reveals the presence of the radioactive substance (arrow).

**Figure 2:**
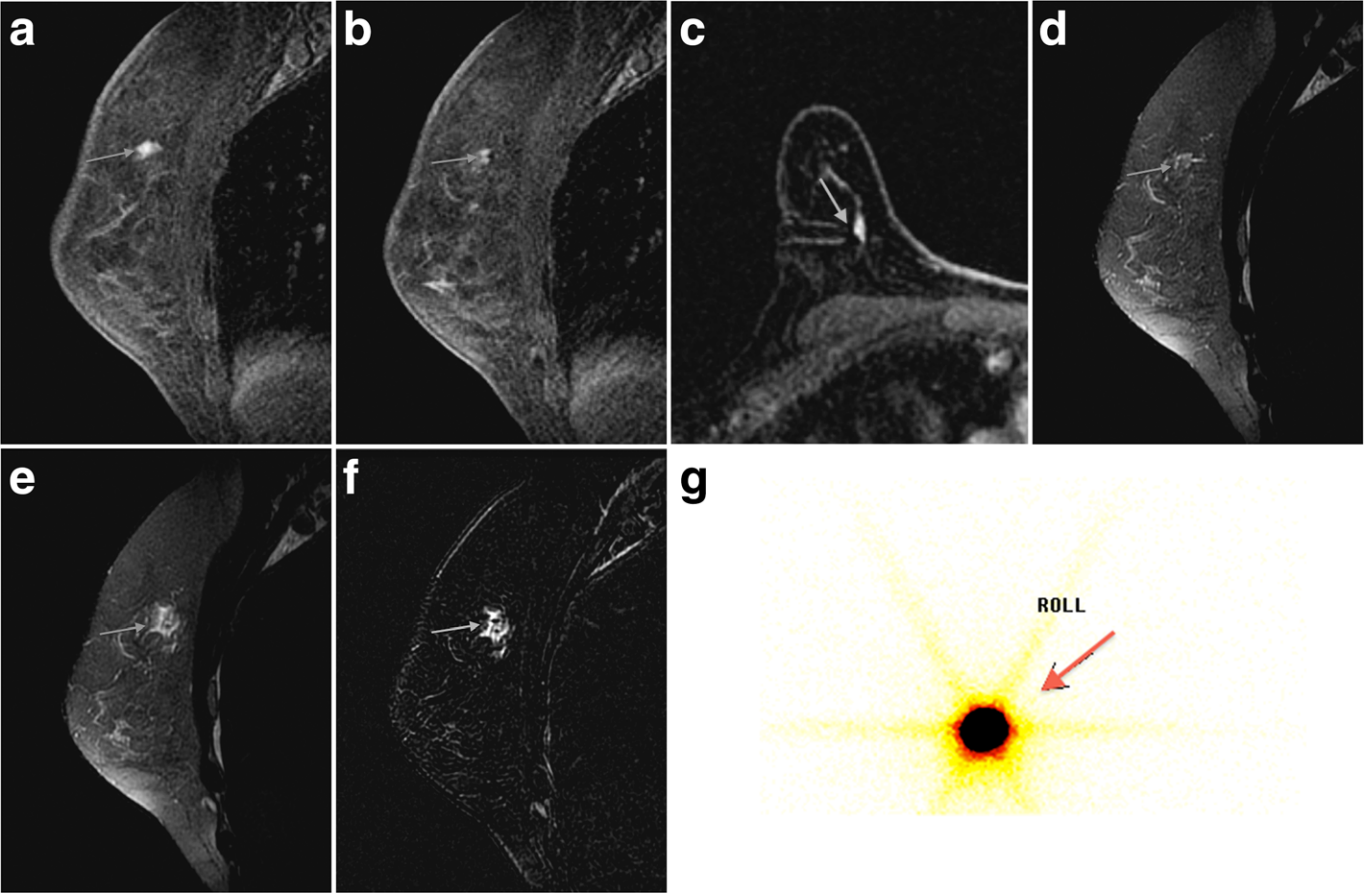
**A 38-year-old woman with invasive ductal carcinoma (IDC) of the right breast. (a)** Sagittal T1-weighted contrast-enhanced sequence shows a focal nonmass enhancement in the upper inner quadrant of the right breast (arrow). **(b)** Sagittal T1-weighted contrast-enhanced sequence reveals the lesion with the needle, low-signal-intensity dot, inside (arrow). **(c)** Axial T1-weighted contrast-enhanced sequence shows the needle tip close to the lesion (arrow) **(d)** Sagittal T2-weighted sequence shows the exact location of the lesion after correlation with the contrast-enhanced sequences (arrow). **(e)** Sagittal fat-suppressed T2-weighted sequence after injection of Tc-99m MAA followed by 1 mL of distilled water reveals a high-signal-intensity area and confirms that the radioactive material is in the exact location of the lesion. **(f)** Sagittal T2-weighted sequence subjected to subtraction technique: the high-signal-intensity area from the water injected can be visualized better (arrow). **(g)** Scintigraphic control reveals the presence of the radioactive substance (arrow).

A total of four malignant lesions (25%), all invasive ductal carcinomas (IDC), were verified upon histopathologic examination. The median size of the malignant lesions was 0.85 cm (range, 0.6-1.2 cm). The first carcinoma was an oval-shaped mass with smooth margins, which presented a plateau curve in the delayed enhancement phase and was classified as BI-RADS 3 (Figure 1). The second, third, and forth carcinomas were non-mass-enhancement masses with linear, focal, and focal distributions, respectively (Figure 2); all three were classified as BI-RADS 4 (Tables 1 and 2).

**Table 1:** Malignant histopathological findings based on patient age, MRI indication, breast density, and MRI lesion characteristics

**Histopathological findings**	**Age**	**MRI indication**	**Breast density**	**Morphological and dynamic characteristics of the lesions**	**Lesion size (cm)**	**BI-RADS**
Invasive ductal carcinoma, grade I	51	Known contralateral breast cancer	Heterogeneously dense	Linear nonmass enhancement	1.2	4
Invasive ductal carcinoma, grade I	78	Known contralateral breast cancer	Heterogeneously dense	Oval shape and smooth margins mass, type 2 curve	0.7	3
Invasive ductal carcinoma, grade II	38	Known ipsilateral breast cancer	Extremely dense	Focal nonmass enhancement	0.6	4
Invasive ductal carcinoma, grade I	54	Problem-solving	Heterogeneously dense	Focal nonmass enhancement	1.0	4

**Table 2:** BI-RADS classification and histopathological findings

	**Malignant**	**Benign**	**High-risk**	**Total**
BI-RADS 3	1	0	1	2
BI-RADS 4	3	9	2	14
Total	4	9	3	16

There were nine benign lesions (56.25%) with the following classifications: fibroadenoma (n = 2), columnar cell alteration without atypia, apocrine metaplasia, adenosis (n = 2), focal florid ductal hyperplasia (n = 2), nodular florid adenosis (n = 1), intramammary lymph node (n = 1), and fat necrosis (n = 1). The median size of the benign lesions was 1.0 cm (range, 0.8-4.0 cm) (Tables 3 and 2).

**Table 3:** Benign histopathological findings based on patient age, MRI indication, breast density, and MRI lesion characteristics

**Histopathological findings**	**Age**	**MRI indication**	**Breast density**	**Morphological and dynamic characteristics of the lesions**	**Lesion size (cm)**	**BI-RADS**
Columnar cell alteration without atypia, apocrine metaplasia, adenosis	57	Integrity of breast implants	Heterogeneously dense	Segmental nonmass enhancement	3.3	4
Columnar cell alteration without atypia, apocrine metaplasia, adenosis	60	Problem-solving	Heterogeneously dense	Ductal nonmass enhancement	1.6	4
Fat necrosis	55	Previous personal history of breast cancer	Scattered fibroglandular densities	Ductal nonmass enhancement	4	4
Intramammary lymph node	53	Occult primary tumor and altered axillary lymph node	Scattered fibroglandular densities	Irregular shape and margins mass, type 3 curve	1	4
Nodular florid adenosis	42	Problem-solving	Heterogeneously dense	Oval shape and smooth margins mass, type 3 curve	1	4
Focal florid ductal hyperplasia	56	Problem-solving	Scattered fibroglandular densities	Focal nonmass enhancement	1.8	4
Focal florid ductal hyperplasia	51	Problem-solving	Scattered fibroglandular densities	Lobulated shape and smooth margins mass, type 3 curve	0.9	4
Fibroadenoma	46	Integrity of breast implants	Scattered fibroglandular densities	Oval shape and smooth margins mass, type 3 curve	1	4
Fibroadenoma	57	Problem-solving	Scattered fibroglandular densities	Oval shape and smooth margins mass, type 2 curve	0.8	4

Additionally, there were three high-risk lesions (23.1%) with the following classifications: intraductal papilloma (n = 1), fibroadenoma with atypia (n = 1), and atypical ductal hyperplasia (n = 1). The median size of the high-risk lesions was 0.6 cm (range, 0.6-2.8 cm) (Tables 4 and 2).

**Table 4:** High-risk histopathological findings according to patient age, MRI indication, breast density, and MRI lesion characteristics

**Histopathological findings**	**Age**	**MRI indication**	**Breast density**	**Morphological and dynamic characteristics of the lesions**	**Lesion size (cm)**	**BI-RADS**
Intraductal papilloma	51	Problem-solving	Heterogeneously dense	Oval shape and smooth margins mass, type 3 curve	0.6	4
Fibroadenoma with atypia	52	Problem-solving	Heterogeneously dense	Oval shape and smooth margins mass, type 2 curve	0.6	3
Atypical ductal hyperplasia	52	Problem-solving	Heterogeneously dense	Ductal nonmass enhancement	2.8	4

After revision, surgical histology and MR imaging findings were considered concordant in all benign and high-risk cases.

The PPV of MRI-ROLL was 25% (4/16). Importantly, the PPV was greatest if the indication for the initial MR examination was a current history of breast cancer (3/4 = 75%). Among the three carcinomas diagnosed on MRI-ROLL in women with synchronous cancer, the cancers that had MRI localization were in the ipsilateral breast in one case and in the contralateral breast in two cases (Table 1).

### Procedure time, complications, accuracy, and follow-up

The median duration time to perform MRI-ROLL was 26 minutes (range, 20–37 minutes). No complications occurred during any of the interventions. However, one case had to be excluded because a hematoma formed during the needle placement and the radioactive substance came back after the injection, requiring a wire placement. Therefore, this study achieved technical success in 16/17 (94.1%) of the cases.

The tip of the needle was placed within the lesions in 12 of the 16 cases (75%) and within 0.3 cm of the edge of the lesions in the remaining four cases (25%) prior to the radioactive material injection. Because the high-signal-intensity area observed on the T2-weighted sequence and on the subtracted images was present in the exact location of the lesion after the water administration and the scintigraphic control, the radioactive material was confirmed to be present in all cases.

Exact and complete lesion removals were confirmed in all cases (100%) that underwent MRI six months postoperatively, i.e., eight of the 16 cases (50%). Four patients (25%) did not have MRIs because their surgeries had been performed less than six months before the end of the study. The remaining four patients (25%) did not undergo MRI because the surgeon did not request it.

## Discussion

Here, we present the technique and results of MRI-ROLL of suspicious breast lesions only observed on MRI. In total, we successfully performed MRI localizations of 16 breast lesions in 15 patients. Although ours was a small series, the findings suggest that this localization technique is rapid, technically successful, and accurate, without the challenges of MRI-guided wire localization.

In our study, the PPV of MRI-ROLL was 25%, which is similar to the PPV of MRI-guided wire localization [7]. The overall ratio of benign to malignant biopsies among MRI-detected lesions ranges between 1:1 and 3:1, depending on diagnostic criteria and indications, e.g., in case of a personal or a family history of breast cancer, invasive procedures are likely to be more frequently indicated [2,11]. In our study, carcinoma was revealed in women with known cancer who had been referred for MRI-guided localization for preoperative staging. Of the four carcinomas identified, one was classified as BI-RADS 3 and three as BI-RADS 4. In addition, the three BI-RADS 4 lesions exhibited morphology and enhancement characteristics that were not specific for malignancy. Even without suspicious characteristics, lesions in a patient with a present history of ipsilateral or contralateral breast cancer should be investigated and biopsied.

Our actual MRI-ROLL time was similar to the 15–59 minute time range that has been previously reported for MRI-guided wire localization [9-11]. With more experience, we expect to perform procedures faster and utilize fewer sequences, while maintaining a high success rate.

Although we did not experience complications during the procedures, we did not succeed in one case and had to exclude it from our study. In that particular case, a hematoma formed during placement of the needle; due to the pressure caused by the hematoma, the radioactive substance could not be injected properly, therefore requiring wire placement. Injection of the radioactive substance may also be challenging when the lesion is harder or more compact.

Currently, the added benefit of MRI in the detection of clinically, mammographically, and ultrasonographically occult cancers is well known. Cancers that are only truly visible on MRI are detected in 14-35% of patients who undergo breast MRI for a variety of reasons [9]. One of the most frequently used methods for sampling MRI-detected lesions remains needle localization with a wire, either because it provides a better approach to breast lesions that are difficult to access or because it is less expensive and more available than other MRI biopsy methods. Although excisional biopsy after MRI-guided wire localization has proven to be a successful method for obtaining adequate material for pathological evaluation, the technique is associated with several disadvantages, including technical difficulties for the interventional radiologist and the surgeon, thereby limiting the success rate of the procedure and increasing the surgery duration time [2].

A potential problem with MRI-guided wire localizations is that the breast is compressed during the deployment of the wire but not during the surgical excision [11]. This deployment creates an accordion effect. The accordion effect has been a recognized explanation for partly excised or missed lesions after MRI-guided needle localizations [2,9,11,22,23]. The accordion effect in MRI-ROLL is not a considerable problem because the radioactive substance is injected on or very close to the lesion and does not change position when the compression is released. Wire displacement or migration between localization and surgery has been recognized in the MRI and mammographic literature [2,9]. MRI-ROLL also solves this problem.

There was no important difference between the cost of the radioactive substance and the titanium wire. However, considering the surgical time, the preoperative wire localization can result in a higher cost to the patient.

Confirmation of lesion retrieval remains an issue in MRI-guided localization with a radioactive marker or a wire. Because the lesion does not enhance *ex vivo*, there is no possibility of direct verification that the correct area has been excised [1]. Correlation of imaging and histology plays an important role in this procedure, as in breast biopsy with any method [10,11,24]. If the pathological assessment yields a benign histology, review of the original MRI and biopsy imaging is advised to reassess the level of suspicion and adequacy of sampling. For cases that are felt to be discordant or possibly missed, repeat biopsy or surgical excision is recommended as soon as can be reasonably tolerated by the patient.

Postoperative MR imaging should be incorporated into the routine follow-up of patients who have MR imaging-guided localization to ensure lesion retrieval if the procedure yields benign findings and to assess for residual tumor if the procedure reveals breast cancer. A study analyzed postoperative MRI scans performed on 33 lesion sites, suggesting that the lesion was completely excised in 29 (87.9%), partly excised in three (9.1%), and missed in one case (3.0%) [11]. Another study reported 13.5% inadequate removal of the lesion despite correct needle positioning [2]. In our study, the exact and complete lesion removal was confirmed in all cases (100%) that underwent six-month follow up MRI. However, postoperative MR imaging was not routinely performed, although it was suggested to the surgeons in the procedure report. To this point, only half of the cases have undergone postoperative MRI.

Our study has some limitations. First, our series is relatively small. The two main reasons for the study’s small size are the following: MRI procedures continue to be expensive, and MRI-ROLL is a new technique that is not well known by mastologists. Further studies should consider increasing the sample number to improve the statistical power. Second, the water injected after the radioactive substance in the target lesion is only observed on MRI. Therefore, it is impossible to determine the exact injection site using any other imaging modality. The same occurs in the single study published in the literature regarding MRI-ROLL, in which the MRI contrast material injected after the radioactive material was only observed on MRI [7]. The scintigraphy identifies the radioactive material but does not reveal the exact injection site. Further work in this area might also include the injection of a radiographically visible marker within the lesion in addition to the injected radioactive substance, e.g., an iodinated contrast or air [25], so that the surgeon can see the location of the lesion with respect to the nipple, the chest wall, and the remainder of the breast tissue using mammography.

## Conclusions

MRI-ROLL offers a relatively rapid and accurate means for performing preoperative MRI-guided localizations in the breast. The results of this preliminary study show that MRI-ROLL is technically feasible and safe. This is particularly important, as radio-guided surgery is easier to perform, allows an effective lesion resection, and leads to better cosmetic results. Larger studies and a comparison between the results of MRI-ROLL and MRI-guided wire localization are needed before this technique can become routine clinical practice.

## Competing interests

No competing interests to disclose.

## Authors’ contributions

FPAP performed the procedures, participated in the design of the study, collected and analyzed the data, and drafted and submitted the manuscript. GM and MJGC performed the procedures and reviewed the manuscript. MVFTO participated in the procedures and reviewed the manuscript. ELG participated in the design and coordination of the study and reviewed the manuscript. LMBF conceived the study, participated in its design and coordination, and approved the final version. All authors have read and approved the final version of this manuscript.

## Authors’ information

LMBF, MD, PhD, Full Professor of Medical School of Federal University of Rio de Janeiro, Head of Department of Radiology of Hospital Clementino Fraga Filho (Federal University of Rio de Janeiro Hospital), Nuclear Medicine Physician of Clínica de Diagnóstico por Imagem and Hospital Samaritano, Rio de Janeiro.

## Pre-publication history

The pre-publication history for this paper can be accessed here:

http://www.biomedcentral.com/1471-2342/13/33/prepub
